# Revealing the source: How awareness alters perceptions of AI and human-generated mental health responses

**DOI:** 10.1016/j.invent.2024.100745

**Published:** 2024-04-27

**Authors:** Gagan Jain, Samridhi Pareek, Per Carlbring

**Affiliations:** a Department of Psychology, Manipal University Jaipur, India; b Department of Psychology, Stockholm University, Stockholm, Sweden

**Keywords:** Chatbots, Chat GPT, Artificial intelligence, Mental health

## Abstract

In mental health care, the integration of artificial intelligence (AI) into internet interventions could significantly improve scalability and accessibility, provided that AI is perceived as being as effective as human professionals. This longitudinal study investigates the comparative perceptions of ChatGPT and human mental health support professionals across three dimensions: authenticity, professionalism, and practicality. Initially, 140 participants evaluated responses from both sources without knowing their origin, revealing that AI-generated responses were rated significantly higher across all dimensions. Six months later, the same cohort (n = 111) reassessed these messages with the source of each response disclosed, aiming to understand the impact of source transparency on perceptions and trust towards AI. The results indicate a shift in perception towards human responses, only in terms of authenticity (Cohen's d = 0.45) and reveal a significant correlation between trust in AI and its practicality rating (r = 0.25), but not with authenticity or professionalism. A comparative analysis between blind and informed evaluations revealed a significant shift in favour of human response ratings (Cohen's d = 0.42–0.57), while AI response ratings experienced minimal variation. These findings highlight the nuanced acceptance and role of AI in mental health support, emphasizing that the disclosure of the response source significantly shapes perceptions and trust in AI-generated assistance.

## Highlights

• AI and human mental health support perceived equally without source disclosure.
• Transparency of AI origins shifts trust and perception favorably towards humans.
• AI's practicality trusted, yet authenticity and professionalism still questioned.
• Study introduces a trust scale correlating AI practicality with user trust levels.
• Findings may influence the integration of AI into mental health practices.

## 1 Introduction

The World Health Organisation (WHO) describes mental health as a state of well-being where individuals recognize their abilities, manage life's stresses, work productively, and contribute to their community (World Health Organisation, 2014). An estimated 29 % of people experience mental disorders in their lifetime, a significant cause of global disability (Steel et al., 2014; World Health Organisation, 2008). Around 70 % of those with mental illnesses globally don't receive formal treatment, due to various reasons including stigma and a perceived lack of need (Wang et al., 2007; Mojtabai et al., 2011; Conner et al., 2010). Access to care is hindered by a shortage of mental health professionals, with developed countries having around 9 psychiatrists per 100,000 people compared to as low as 0.1 in low-income countries, making traditional mental health interventions difficult to deliver (Thomas et al., 2009; Cunningham, 2009; Murray et al., 2012; Oladeji and Gureje, 2016; Vaidyam et al., 2019). The WHO highlights that 55 % of individuals in developed countries and 85 % in developing ones lack access to mental health services, exacerbating issues like suicidal behavior (Anthes, 2016; Hester, 2017).

The COVID pandemic also triggered increased demand for mental health services, creating a ‘perfect storm’ of reduced resources and heightened need, leading to a spike in unmet mental health services globally (World Health Organisation, 2020). Recent research indicates a surge in the unmet need for psychotherapy and counseling during the pandemic (De Witte et al., 2021; Kaiser Family Foundation, 2021; Van Daele et al., 2022).

### 1.1 Chatbots and mental health

To combat the shortage of mental health professionals, technology, including mobile health apps and chatbots, is being employed (Lucas et al., 2017; Inkster et al., 2018; Seiferth et al., 2023; Smith et al., 2023). A chatbot is a system that is able to converse and interact with human users using spoken, written, and visual languages (Vaidyam et al., 2019; Kumar et al., 2016). Chatbots are seen as a promising tool to expand access to mental health interventions. These interactive platforms may be particularly beneficial for individuals who have previously been hesitant to seek mental health support, often due to concerns about being stigmatized (Vaidyam et al., 2019; Lucas et al., 2017). These innovative technologies leverage artificial intelligence to provide therapeutic interactions, resembling those with a human therapist (Carlbring et al., 2023). Chatbots like Woebot, developed by researchers at Stanford University, use cognitive-behavioral techniques to assist users in managing mental health conditions such as depression and anxiety (Fitzpatrick et al., 2017). Similarly, Wysa, designed with an empathetic conversational style, offers evidence-based support and has been evaluated for its effectiveness in improving mental well-being (Inkster et al., 2018). Another notable example is Tess, a psychological artificial intelligence chatbot, which has been shown to reduce depression symptoms significantly in users (Fulmer et al., 2018). The growing evidence base for these digital interventions highlights their potential in expanding access to mental health care, particularly in areas with a shortage of mental health professionals (Vaidyam et al., 2019). However, it is important to acknowledge that while chatbots can provide immediate, scalable support, they are not a replacement for traditional therapy but rather a complement to existing mental health services.

### 1.2 Trust and mental health chatbots

Establishing trust between mental health chatbots and their users is a crucial aspect of their effectiveness, but it poses significant challenges. Trust in these digital platforms hinges on their ability to provide reliable and empathetic interactions, mirroring the nuanced communication of human therapists. However, building this level of trust is complicated by the inherent limitations of chatbots, such as their inability to comprehend complex human emotions and the nuances of mental health issues fully (Luxton, 2015). Privacy concerns also play a significant role; users must feel confident that their personal and sensitive data are secure and handled appropriately. Despite these challenges, some studies indicate a growing acceptance and trust in mental health chatbots, attributed to their consistent availability and the anonymity they offer, which can be particularly appealing to individuals who might otherwise avoid seeking help due to stigma (Vaidyam et al., 2019). However, ensuring the ethical use of data and the continual improvement of AI algorithms to better understand and respond to human emotions remains a pivotal challenge in strengthening the trust between users and mental health chatbots (Miner et al., 2020).

### 1.3 Study objective

Building on our previous exploration of GPT-3.5, one of the most advanced Natural Language Processing (NLP) technologies, this follow-up study aims to understand the perception dynamics of AI-generated responses in the realm of mental health support for young people. This study may relate more to well-being issues rather than actual mental health conditions where AI may not have access to data to produce or be able to give appropriate responses.

In the first phase, we compared the responses of Chat GPT (using 1–5 star rating for each of the responses) with the responses of Master's level Clinical psychology students who have been exposed to various internships in Clinical psychology. The responses were compared in the dimensions of authenticity, professionalism, and practicality, without revealing the source of these responses to the participants. The purpose was, rather, to simply understand the differences a lay person would find in ChatGPT vs Human responses. They were under the impression that all responses originated from human counselors, thus providing an unbiased assessment of AI's capabilities in mimicking human-like support (Lopes et al., 2023).

Now, six months later, with the same set of problems, responses, and participants, we introduce a pivotal variable – awareness. Participants are now informed about the source of each response, distinguishing between those generated by humans and those by ChatGPT. This study aims to juxtapose the findings of the previous research with the current results, focusing on the comparative analysis of the ratings in practicality, authenticity, and professionalism for both ChatGPT and human responses. Furthermore, an additional dimension of this study is the measurement of trust in AI. By employing a trust scale, we aimed to correlate the levels of trust participants have in robotic systems with the ratings they assign to ChatGPT's responses. This comprehensive approach offers a nuanced understanding of how awareness of an AI source influences the perception and trust in the responses provided by such advanced technologies in a sensitive domain like mental health support.

## 2 Methodology

This study conducts a detailed analysis to explore the effects of disclosing the AI origin on young adults' perceptions of mental health support services. Six months after our initial study, which compared responses from ChatGPT and human professionals without revealing their origins, we reintroduced the same set of problems, responses to the same set of participants. This time, however, we clearly distinguished between responses generated by humans and ChatGPT and briefed our participants about the same. The core of our methodology revolves around a comparative analysis. Participants were now informed whether the responses they will evaluate are from AI or humans. The focus is on reassessing the practicality, authenticity, and professionalism of each response, contrasting these findings with those from the previous blind study.

Moreover, we also measured the trust on robots using Human-Robot Interaction Trust Scale (HRITS) scale (Pinto et al., 2022). There are total 11 items in this scale which is designed to measure participants' trust in AI and robotic systems using *5- point Lickert scale* from strongly disagree (1) to strongly agree (5). We aim to draw correlations between the trust levels participants have in these systems and the ratings they assign to ChatGPT's responses. This dual approach of comparing the perception of AI generated responses versus Human responses and calculating the level of trust on robots, offers a nuanced insight into the impact of source awareness on perceptions of AI-generated support in the sensitive field of mental health. By doing so, we hope to shed light on the evolving role and acceptance of AI technologies in contexts that have traditionally been the domain of human professionals.

### 2.1 Sample

In the first phase of the study a combination of convenience and purposive sampling was used with a total of 140 participants (101 female, 37 male and 2 opted not to provide information about their gender) aged between 18 and 43 (SD = 3.444). Participants had various educational backgrounds, including undergraduate and graduate degrees in psychology, as well as degrees in architecture, business, law, education, microbiology, and computer science. Convenience sampling was employed through the distribution of online forms and in-person visits to classrooms, which allowed recruitment of potential participants who were available and willing to participate.

For the follow-up phase, we reconnected with the initial cohort and obtained participation from 111 individuals (82 females and 29 males), aged between 18 and 43 years (M = 25, SD = 3.5). The continuity of participants allows for a comprehensive longitudinal analysis.

### 2.2 Data collection

#### 2.2.1 Study-I

The study began with a “Listening Circle” activity, where participants shared distressing questions or situations in their lives. These were collected via paper-pencil surveys and redistributed randomly among participants for solution suggestions. Out of 50 participant- generated questions, 10 open-ended ones were chosen, covering areas like interpersonal issues, stress, and intrapersonal conflicts, typical of mental health support scenarios. Participants evaluated two types of responses for each question, one from ChatGPT and one human- generated, in a single-blind format where AI origins were unknown. The presentation order of responses was counterbalanced across participants using Google Forms to control for order effects and eliminate bias. The responses, similar in length but not identical, were rated on a 5- point Likert scale for authenticity, professionalism, and practicality. Data was gathered through self-report forms and securely stored, including participants' demographics, question numbers, and ratings.

### 2.3 Sample problem statement and response

***Problem statement***: *I feel very unproductive and that I lack motivation to do things. I have a lot of things planned but I fail to execute them.*

**Human Response**: My advice to tackle this would be to start with the smallest of things and reward yourself for doing it. Set a very small goal at first, it can be anything like just waking up an hour earlier than you usually do or just giving an extra of 15 min to your studies or any of your hobbies. And whenever you accomplish that reward yourself. That'll give you a kind of satisfaction that treating yourself on a normal day doesn't and you'll find yourself getting addicted to it. And always remember that being productive is not only physical work or academics. There's social and mental productivity too. Work on that consciously and everything else will eventually fall into place.

**AI Response**: Feeling unproductive and lacking motivation can be frustrating and can make it difficult to accomplish things that you have planned. It can be helpful to break down goals into smaller, manageable tasks and set achievable goals for each task. Additionally, finding ways to increase motivation such as finding purpose, creating a positive environment, or finding accountability can help. Identifying any underlying issues that may be contributing to lack of motivation, such as stress or depression, and seeking support from a therapist or counselor can also be beneficial. Lastly, being kind to yourself and celebrating small successes can help increase productivity and achieve goals.

### 2.4 Procedure

The questions that were distributed to the participants were the same as used in the previous study but this time with a difference that the participants had information regarding which set of responses were generated by humans and which by AI. The time lag ensured that the participants did not choose their responses on the basis of confirmatory bias as six months would fade away the traces of any previous responses (Wixted, 2004; Rubin and Wenzel, 1996). The participants then had to rate the responses on the basis of authenticity, professionalism and practicality as mentioned along with knowledge of the source of response. We also used a trust scale to measure how comfortable participants were with things like their data being safe and about their perception of utility and effectiveness of therapy. The responses were collected using an online survey and the data was then compiled for further study.

### 2.5 Ethical considerations

Prior to their participation in the study, informed consent was obtained from each participant. Participants were given an information sheet as part of the survey form, which outlined the purpose and procedures of the study. Additionally, participants were assured that there would be no data personalization, as their identities were not used in generating responses from either the human or the AI. They were explicitly informed that no personal sensitive data would be collected, and the study did not aim to influence or manipulate them in any way. Participants were also informed that their participation was voluntary, and they had the right to withdraw from the study at any time without facing any penalty. All data collected was kept confidential and anonymous, ensuring complete privacy and data protection. The study was duly approved by the Ethics committee of the Department of Psychology, Manipal University Jaipur. Although proper care was taken that no triggering problem was used in the survey, the participants were also informed about the counseling services available at our university if the need arose. As the responses were circulated to experts, no such comment which was rejected on this basis (misleading or harmful) was included in the study.

### 2.6 Statistical analysis

The data collected on the sample of 111 participants was further analysed for any significant differences in the ratings for authenticity, professionalism, and practicality. Since perceptions regarding AI responses were to be checked in comparison with human responses, a *t*-test was employed comparing the differences in the Human versus AI responses across these three dimensions. Further a correlation was run to find out if trust on robots was correlated with the chat GPT responses. The results were then interpreted on the basis of responses received. In addition to that the paired t-test was also employed to evaluate the differences between AI and human ratings on all the three dimensions. Additionally, since only 111 of the 140 participants took part in the second round of the study, it was challenging to assess participant congruity because some chose to remain anonymous. The primary focus of the study was to determine if there was a statistical shift in the participants' perceptions as a group; therefore, individual analyses were not conducted.

## 3 Result

Table 1 presents the overall mean ratings for authenticity, professionalism, and practicality in responses generated by both ChatGPT and humans, along with the outcomes from a paired samples t-test conducted to compare these ratings. While the mean rating for authenticity was higher for human responses (37.66) compared to ChatGPT (34.85), this difference was statistically significant, suggesting participants perceived human interactions as more genuine and sincere. Notably, this contrasts with our previous finding where ChatGPT was rated higher for authenticity when the source was not disclosed. For professionalism, the mean rating was slightly higher for ChatGPT (36.85) than for humans (35.83); however, this difference was not statistically significant, indicating no substantial difference in perceived competence and skill between ChatGPT and human therapists. Similarly, for practicality, although human responses scored slightly higher (37.05) compared to ChatGPT (36.24), this difference was also not statistically significant. This suggests that the convenience and utility of the methods used by human therapists were perceived to be comparable to those of ChatGPT. Overall, these results indicate that the only domain in which participants' perceptions differed significantly when the source was revealed was authenticity, favoring human responses.

**Table 1:** Comparative mean ratings and paired samples *t*-test for ChatGPT-generated and human- generated responses on authenticity, professionalism, and practicality.

Dimension	AI responses	Human response	*t(111) Cohen's d*
	Mean (SD)	Mean (SD)	
Authenticity	34.85 (6.15)	37.66 (6.36)	4.233⁎ 0.45
Professionalism	36.85 (6.70)	35.83 (6.43)	1.317 0.16
Practicality	36.24 (6.44)	37.05 (6.38)	1.138 0.13

⁎ Significant at p < .01.

As the correlation table suggests, the trust in robots scale was found to be significantly correlated with practicality at 0.01 level of significance while there was no significant correlation of the said scale with authenticity and professionalism.

Table 3 compares the means of blind evaluation and informed evaluation in terms of statistical significance in the differences of the ratings of responses in all the three domains viz. authenticity, professionalism, and practicality. The results indicate that while the mean differences in blind and informed evaluations were not found to be statistically significant for AI in terms of authenticity, professionalism, and practicality, all the three domains showed a statistically high rating for human responses. This indicates that the perception for AI responses remained the same, while the perception for authenticity, professionalism and practicality grew highly in favour of human response as compared blind evaluation. The mean differences in authenticity and professionalism in human responses are extremely statistically significant at.0001 level while that of practicality is very statistically significant at p value of 0.001.

## 4 Discussion

The current study aimed at understanding the general perception of the sample population towards AI and human generated responses against various emotional issues. In continuation to the first phase of the study the same cohort was asked to rate the solution responses in various situations. The responses were either generated by Humans or those by ChatGPT, only, that unlike previous study, this time the source of responses was revealed. While the previous study attempted to assess the competency of AI in comparison to humans for generating responses to emotional problems, this study went a step ahead to assess perception of humans towards AI by reveling the source of responses as well as by applying the trust scale. Indeed, the same set of responses revealed a change in ratings as compared to the previous study, indicating that though the content was the same, how the content was perceived was changed.

The results (Table 1) revealed a higher mean rating for human responses in terms of authenticity and practicality but the results for practicality were not statistically significant. This was a change from the previous trend where chat GPT was rated significantly higher in both. In terms of professionalism the results were in line with the previous study and ChatGPT received a higher rating. While these all dimensions received a statistically significant favorable response in the previous study, in the current study the differences were statistically significant only for authenticity in favour of humans.

This significantly higher score of human responses for authenticity could be explained by the intrinsic human capacity for empathy and emotional intelligence which involves a profound sense of mutual and nuanced understanding and emotional depth that AI systems like ChatGPT currently struggles to achieve (Perry, 2023). The higher mean ratings on practicality for human signifies the importance of contextual understanding. Humans naturally excel in understanding and adapting to complex, nuanced contexts, a skill that is crucial for providing practical advice. Unlike AI, humans can draw from a rich reservoir of personal experiences and social understandings. Human responses are often more tailored to the individual's specific circumstances, reflecting a deeper understanding of their unique situation, needs, and emotions. In fact, in the present study human responses were selected on the basis of face validity by consulting fellow psychologists. On the other hand the higher mean ratings for AI generated responses than human responses in terms of professionalism could be due to AI's ability to deliver factually correct and relevant information efficiently. This aligns with the view that while AI can process and provide information effectively, it may not replace the nuanced understanding and empathy of human interactions. The concept of patient belief influencing treatment outcomes is highlighted in a study by Faria et al. (2017) where the only variable was the patients' belief about the nature of their treatment. Despite all patients receiving the same SSRI, those who thought they were receiving an active placebo showed significantly different outcomes compared to those who believed they were receiving genuine medication (14 % vs 50 %). This emphasizes the crucial role of perception and belief, similar to the impact of source awareness in AI versus human- generated mental health responses.

The same sample was further given a test for trust on robots and it showed a statistically significant correlation only with practicality (Table 2). The correlation for authenticity and professionalism were weak. This lack of correlation between trust on robots and AI ratings can be understood through the anthropomorphic form of commitment between the truster and the trustee (Sweeney, 2023). Holton (1994) suggests that trust involves a specific type of reliance, where betrayal is felt if expectations are not met, contrasting with mere disappointment from machine failure. Hardin (2002) adds a rationality angle, proposing that trust involves assessing risks and expecting the trusted to align their interests with ours for mutual benefit. This concept contrasts with inanimate objects like ladders, which cannot commit, understand reliance, or reciprocate interests, thereby making the concept of trust inapplicable to them. Trust, in this view, requires awareness and a degree of reciprocal understanding, absent in non-sentient entities.

**Table 2:** Correlation between trust in robots scale and perceptions of AI responses in mental health support: authenticity, professionalism, and practicality.

Dimension	*M*	(SD)	*Correlation (trust scale)*	*p*
Authenticity	34.85	(6.15)	0.178	0.061
Professionalism	36.85	(6.7)	0.075	0.431
Practicality	36.24	(6.44)	0.248	0.008

**Table 3:** Comparative Analysis of Perceptions in Study 1 and Study 2 using paired t-test: Evaluating Changes in Authenticity, Professionalism, and Practicality Ratings for AI and Human Responses.

	Chat GPT			p	Human responses			p
	Mean	SD	*t*		Mean	SD	*t*	
Authenticity Blind evaluation (n = 140) Informed evaluation (n = 111)	36.33	7.44	1.69	0.0922	33.72	7.56	4.391	0.0001
	34.85	6.15			37.66	6.36		
Professionalism Blind evaluation (n = 140) Informed evaluation (n = 111)	36.18	7.7	0.7226	0.0470	32.26	7.67	3.923	0.0001
	36.85	6.7			35.83	6.43		
Practicality Blind evaluation (n = 140) Informed evaluation (n = 111)	36.73	7.7	0.532	0.5948	34.02	7.99	3.258	0.0013
	36.24	6.44			37.05	6.38		

The comparison between Study 1 and Study 2 reveals a significant shift in perceptions favoring human responses over AI in terms of authenticity, professionalism, and practicality. Understanding these differences in emotions and perceptions towards human responses reveals a great source of biases that form a part of human perception. This difference in perception of human ratings indicate a clear sense of affinity bias wherein there is a tendency to favour individuals or situations based on shared similarity, experiences etc. (Nikolopoulou, 2023). Artificial intelligence has long been in question due to the extremeness of unfamiliarity (Ye et al., 2019). Additionally, since the responses generated by AI had a complete lack of human touch i.e. without human validation, written and validated by professional psychologists this might also added value to the human generated responses. Despite AI's advantages in precision and efficiency, its lack of human emotional experiences positions it as an ‘unknown’ competitor, often perceived as a threat due to its unfamiliarity (Caporusso, 2023; Gupta et al., 2023). However, when it comes to humans, though divided by so many factors, we are united by humanity or at least humanness. And when this whole humanness is competing with AI, the ratings preferred in the arena of humanness can be easily justified. The findings related to change in perception can also be comprehensively explained through a synthesis of empathy, trust, and psychological attachment theories. Empathy in human interactions plays a pivotal role, as it's a quality deeply ingrained in human nature, allowing for more nuanced understanding and sharing of feelings. This aspect significantly contributes to the higher perceptions of authenticity and practicality in human responses (Elliott et al., 2011; Elliott et al., 2018). On the other hand, trust in AI, a key factor in its acceptance and effectiveness, tends to fluctuate. Initially, users might trust AI for its technical expertise, but over time, they may gravitate more towards human responses for more empathetic and personalized interactions (Blease et al., 2020; Doraiswamy et al., 2020). This evolving trust can explain the decreased ratings for AI in authenticity in the follow-up study. Moreover, the psychological theory of attachment might also influence these perceptions. People generally develop more trust and reliability in entities they are more familiar with, like other humans. This phenomenon could explain why human responses were rated higher in the follow-up study, reflecting a stronger psychological attachment (Riess, 2017).

AI's role in mental health support is increasingly prominent but is often perceived as supplementary to human interaction. The consistent ratings of AI in terms of professionalism and practicability might reflect its perceived role as a reliable and professional tool, albeit not as empathetic as human interactions (Sharma et al., 2020). Even the well tested chatbots like Wysa's content and conversational tools are vetted by a scientific advisory board, ensuring that all interactions are not only technically sound but also align with evidence-based psychological principles. This advisory board likely plays a critical role in maintaining the evidence-based quality of the dialogues, regardless of whether they are hand-crafted or generated through NLP techniques (Inkster et al., 2018).

Human responses were rated significantly higher in professionalism and practicality compared to ChatGPT, with a notable increase in perceived professionalism when the source was disclosed, achieving statistical significance at the 0.0001 level. The source credibility theory suggests that the revealed human source of the responses likely enhanced their perceived credibility and professionalism (Hovland et al., 1953). The comparison between human and AI communication also plays a role; the inherent bias towards human communication being more nuanced and context-aware might lead to a higher appreciation of professional nuances in human responses (Castelo et al., 2019). Additionally, confirmation bias, where people favour information that aligns with their pre-existing beliefs, might lead participants to rate human responses higher in professionalism, and practicality especially if they already believe humans to be more professional in such contexts (Nickerson, 1998).

In summary, this difference between the ratings of study 1 and follow up study attributed to various psychological factors which play a crucial role in shaping perceptions, particularly in scenarios where the source of information significantly impacts its reception.

### 4.1 Limitations

The study's limitations are primarily centered around the representativeness and size of the participant group, which may impact the generalizability of the findings. Additionally, the awareness among participants that they were interacting with AI might have influenced their responses, potentially introducing bias. The limitations of AI's emotional intelligence and its ability to comprehend complex human emotions also pose significant constraints. Prior experience of the study participants with AI has not been considered. There might be a possibility that familiarity with AI may impact the perceptions of the participants regarding the generated responses. Lastly, concerns regarding data privacy and trustworthiness of AI systems in handling sensitive information were notable.

## 5 Conclusion

The study concluded that awareness of AI involvement alters user perception of the interactions, with human responses generally perceived as more authentic and practical compared to AI. This highlights the irreplaceable value of human empathy in mental health support. Although AI is recognized for its professional and informative role, it is seen as a supplement rather than a replacement for human interaction in mental health services. The trust in AI was notably correlated with its perceived practicality, underlining AI's role as a technical tool rather than an emotional support system.

## Declaration of competing interest

The authors declare that there are no conflicts of interest.
